# Chondromyxoid Fibroma: A Rare Case Report and Review of Literature

**DOI:** 10.7759/cureus.803

**Published:** 2016-09-23

**Authors:** Rishit Soni, Chirag Kapoor, Malkesh Shah, Jay Turakhiya, Paresh Golwala

**Affiliations:** 1 Orthopaedics, Sumandeep Vidyapeeth, Vadodara, Gujarat

**Keywords:** chondromyxoid fibroma, benign, bone graft, en bloc resection

## Abstract

Chondromyxoid fibroma (CMF) is one of the rarest benign tumors of cartilaginous origin. It accounts for less than 0.5% of bone tumors and less than two percent of benign bone tumors. It is composed of a mixture of chondroid, myxoid, and fibrous tissues. The diagnosis of CMF depends upon its characteristic histological appearance like a lobular pattern with stellate-shaped cells in a myxoid or chondroid background. We present a case of juxtacortical CMF in a 15-year-old male involving the proximal end of the tibia, which was treated with en bloc excision and bone grafting with excellent results on final follow-up.

## Introduction

Chondromyxoid fibroma (CMF) is one of the rarest benign tumors of cartilaginous origin. It accounts for less than 0.5% of bone tumors and less than two percent of benign bone tumors [1-2]. It mainly affects the metaphysis of long bones, the proximal tibia being the most common location. It mostly occurs in patients who are 10 to 30 years old and presents more commonly in men than in women [1, 3]. It is composed of a mixture of chondroid, myxoid, and fibrous tissues [1]. There are various treatment options for this condition, which include curettage alone, curettage with phenol, and en bloc resection with bone grafting [3]. We present a case of a juxtacortical CMF in a young male patient involving the proximal end of the tibia and its management. Informed consent was obtained from the patient for this study.

## Case presentation

A 15-year-old male patient presented with a four-month history of pain at the proximal end of the right tibia. The pain was a dull ache, mild in intensity without any diurnal variation. A physical examination revealed mild tenderness locally with overlying normal skin, unaffected knee joint movements, and no swelling. The laboratory investigations were within normal limits.

**Figure 1:**
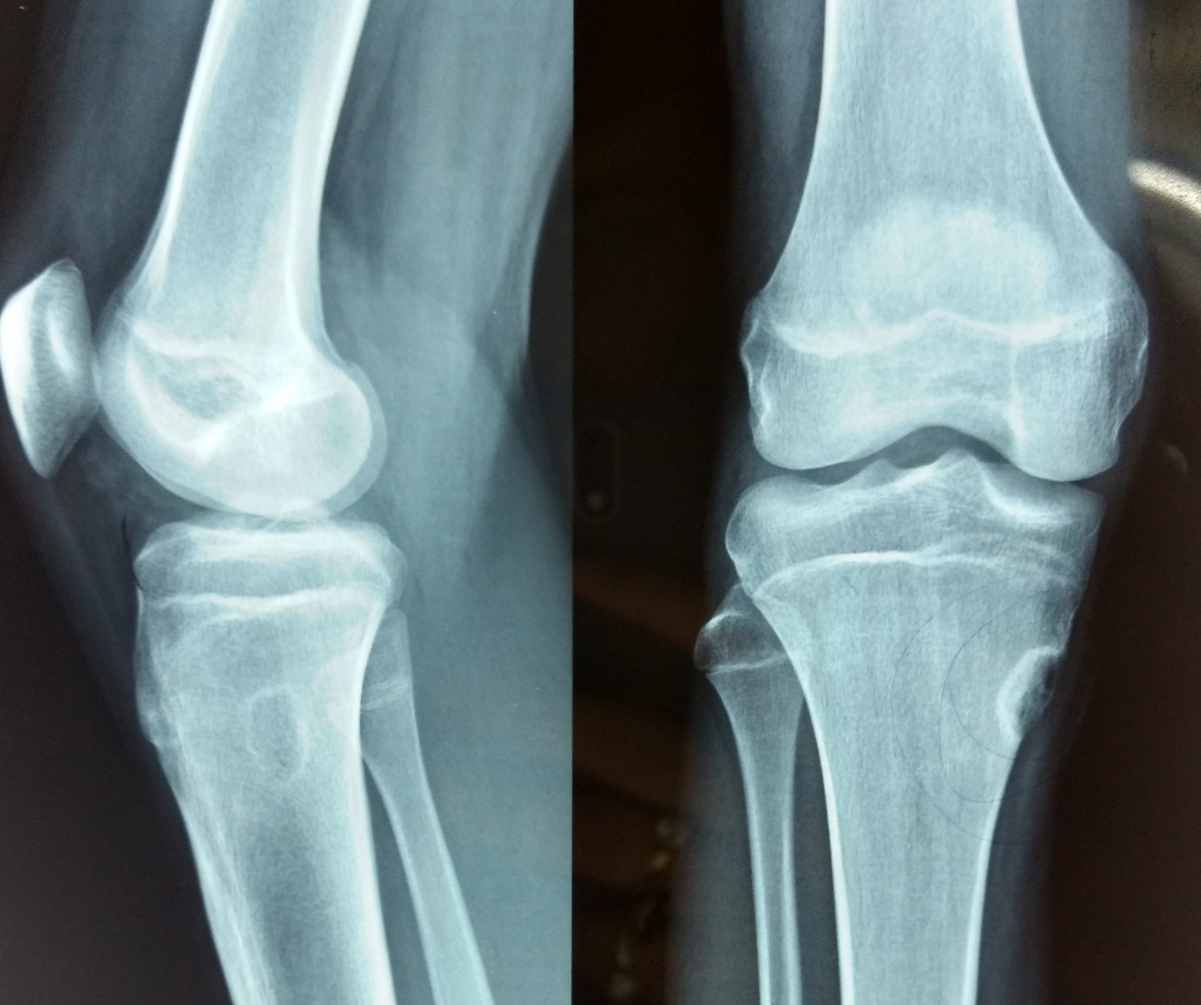
Pre-op radiograph (anteroposterior and lateral views) The images show the lesion in the metaphyseal area of the tibia on the medial aspect.

A radiographic examination showed an osteolytic, radiolucent, eccentric lesion in the metaphysis at the medial border of the proximal tibia, with sclerotic margin and septations with no evidence of periosteal new bone formation [Figure 1].

**Figure 2:**
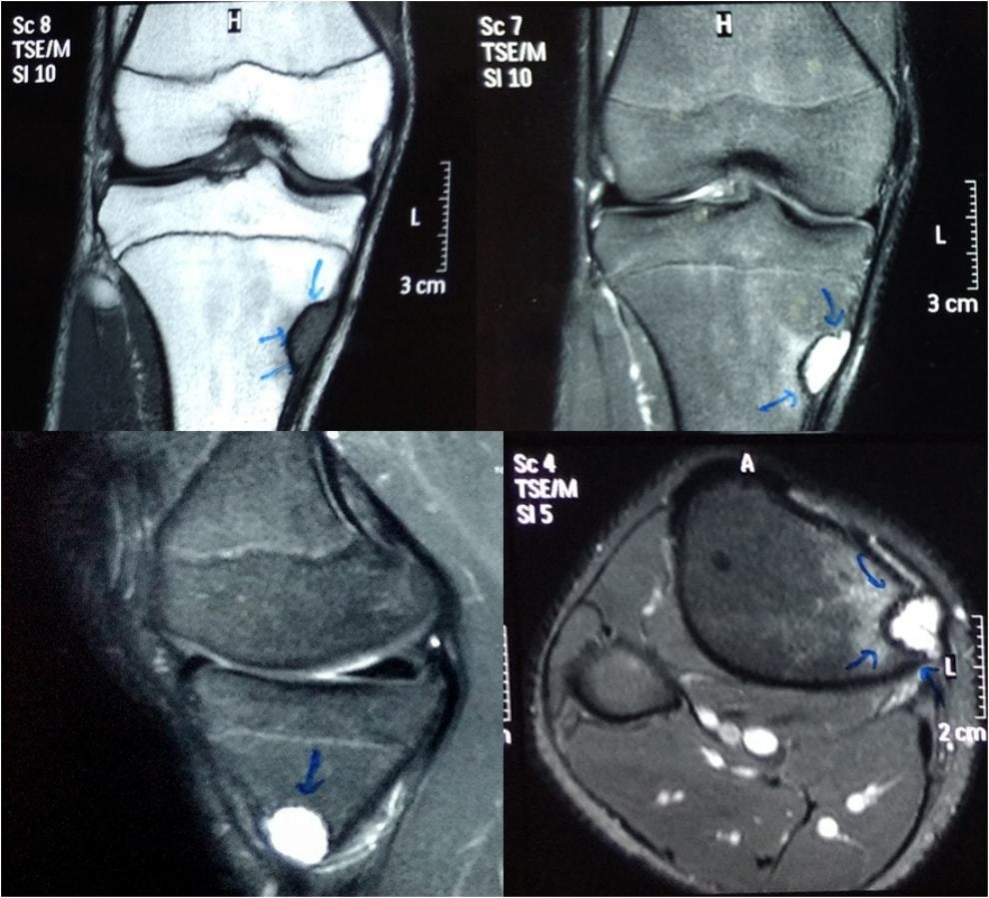
MRI images of the site of the lesion The images show hypointense on T1 and hyperintense on T2.

A magnetic resonance imaging (MRI) scan showed low signal intensity on T1-weighted images at the site of the lesion and heterogeneous, high signal intensity on T2-weighted images suggestive of chondromyxoid fibroma [Figure 2].

**Figure 3:**
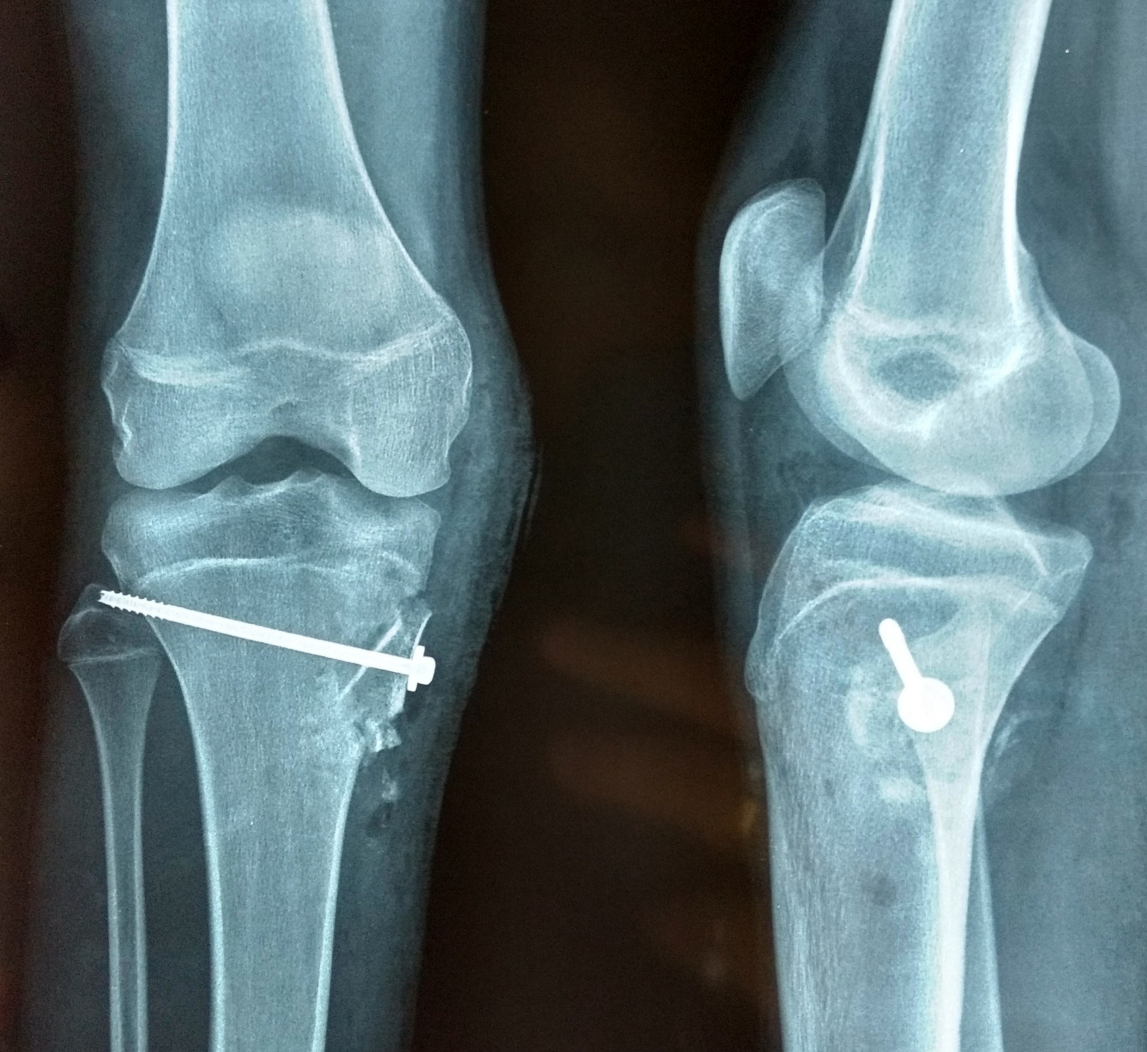
Postoperative radiographs The images show excision of the lesion and the cavity filled with bone graft and fixed with a screw.

After obtaining written consent from the patient, an en bloc excision along with an autogenous tricortical bone graft taken from the ipsilateral iliac bone was performed. The graft was fixed with a cancellous-cannulated screw [Figure 3], and the excised lesion was sent for histopathological examination.

**Figure 4:**
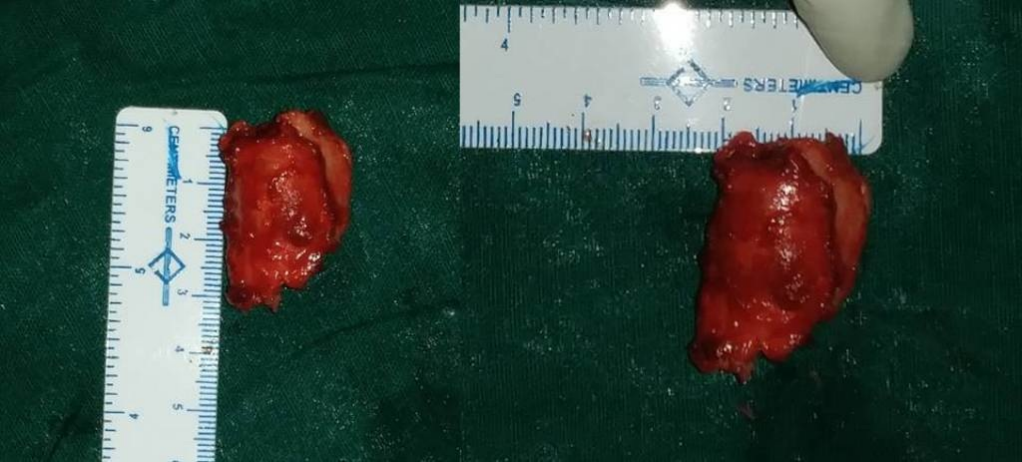
Intraoperative finding The image shows the excised lesion and its size.

**Figure 5:**
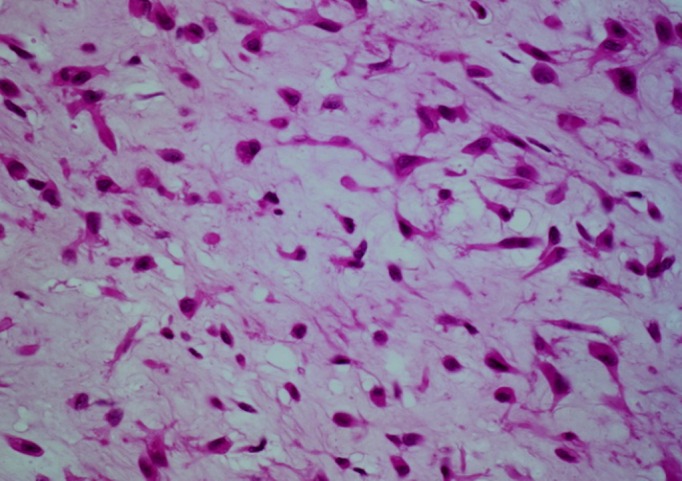
Histopathology slide The image shows fibrocollagenous stroma and myxochondroid islands with stellate-shaped cells.

A gross examination revealed a cartilaginous bony tissue approximately 3.5 x 2 x 1 cm in size with a few hemorrhagic bits [Figure 4]. A histopathological examination of multiple serial sections showed bony tissue with fibrocollagenous stroma and myxochondroid islands with stellate-shaped cells, which confirmed the diagnosis of CMF [Figure 5].

**Figure 6:**
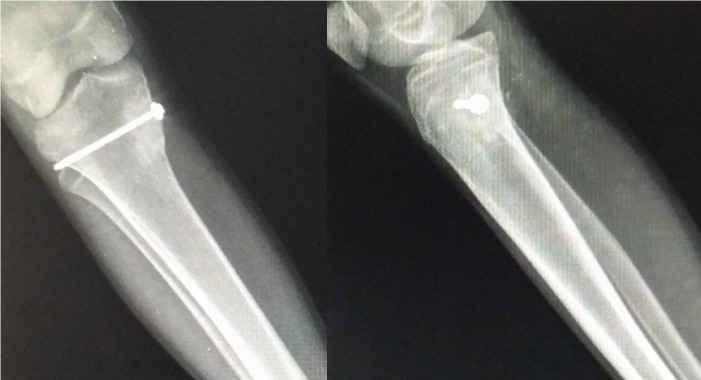
Follow-up radiograph at one year The images show good incorporation of the graft and no signs of recurrence.

Postoperatively, the patient was immobilized in a cylindrical plaster cast followed by partial weight-bearing at six weeks and full weight-bearing at 10 weeks. The graft got incorporated at five months postoperatively, and at the one-year follow-up there were no signs of recurrence [Figure 6].

## Discussion

Chondromyxoid fibroma (CMF) is a benign, locally aggressive tumor of cartilaginous origin and accounts for less than 0.5% of all bone tumors [1]. In 1948, the tumor was first described by Jaffe and Lichtenstein as a lesion derived from cartilage-forming tissue and composed of various proportions of chondroid, fibrous, and myxoid tissues [3]. The common site of the tumor is the metaphysis adjacent to the epiphyseal growth plate, which reinforces the hypothesis that the tumor arises from the remnants of cartilage at these sites [4]. For establishing the diagnosis, a thorough clinical, radiological, and pathological examination is important as it might easily be misdiagnosed as other malignant tumors such as chondrosarcoma because of some pathological similarities [3].

Patients typically complain of pain and swelling at the site of the lesion [1]. The pain is usually mild, intermittent, and a dull ache as seen in our case. If the tumor is located on rare sites like hands or feet, then painless swelling may be the presenting complaint. On some occasions, the tumor may be asymptomatic and may present as an incidental finding on radiographic examination [5].

The radiographic picture shows an eccentrically located lesion in a large limb bone, with an internal well defined scalloped border of sclerotic bone, which was also seen in our case [2]. When the tumor involves a smaller tubular bone like the rib or the fibula, the radiographic appearance is not typical because the lesion may extend throughout the entire width of the affected bone, expanding both surfaces and often making diagnosis between fibrous dysplasia and chondroma difficult [6]. An MRI examination helps in knowing the extent of the spread of the tumor [7].

Diagnosis of CMF basically depends on its characteristic histological appearance. The typical histological features of CMF are a lobular pattern with stellate-shaped cells in a myxoid or chondroid background with hypocellular centres and hypercellular peripheries. Osteoclast-like giant cells are often present at the lobular peripheries [8]. Dahlin stressed that giant cells at the periphery of the chondroid lobules with plump hyperchromatic nuclei with nuclear atypism are characteristic of CMF [9]. Similar features were seen in our case. The differential diagnosis of CMF includes chondroblastoma, chondrosarcoma, enchondroma, and aneurysmal bone cyst, but it is the salient histological features that distinguish these lesions [8].

The treatment options of CMF include simple curettage, curettage with phenol application, and en bloc resection with bone grafting [8]. The tendency to local recurrence after initial curettage seems to be even higher in young children, i.e. 80% [8]. But curettage with phenol application followed by bone grafting has a very low rate of recurrence of seven percent [7]. Further reduction in recurrence rate was observed when the lesion was treated with en bloc excision and bone grafting [6]. Scaglietti, et al. drew attention to the locally aggressive behavior of this tumor in the young and suggested a more radical form of local resection in its management [7]. So, our patient was treated by en bloc excision with tricortical bone grafting, which got incorporated very well, and the lesion has shown no signs of recurrence.

## Conclusions

Chondromyxoid fibroma is an uncommon benign bone neoplasm. It is often confused with other tumors as its pathologic identity is often confused with more aggressive tumors and is misdiagnosed many times. So, proper diagnosis and subsequent management is required for this tumor.
